# Fast Healthcare Interoperability Resources (FHIR) for Interoperability in Health Research: Systematic Review

**DOI:** 10.2196/35724

**Published:** 2022-07-19

**Authors:** Carina Nina Vorisek, Moritz Lehne, Sophie Anne Ines Klopfenstein, Paula Josephine Mayer, Alexander Bartschke, Thomas Haese, Sylvia Thun

**Affiliations:** 1 Core Facility Digital Medicine and Interoperability Berlin Institute of Health at Charité – Universitätsmedizin Berlin Berlin Germany; 2 Institute for Medical Informatics Charité – Universitätsmedizin Berlin Berlin Germany

**Keywords:** Fast Healthcare Interoperability Resources, FHIR, interoperability, health research, health care, health information technology, research, clinical research, public health, epidemiology

## Abstract

**Background:**

The standard Fast Healthcare Interoperability Resources (FHIR) is widely used in health information technology. However, its use as a standard for health research is still less prevalent. To use existing data sources more efficiently for health research, data interoperability becomes increasingly important. FHIR provides solutions by offering resource domains such as “Public Health & Research” and “Evidence-Based Medicine” while using already established web technologies. Therefore, FHIR could help standardize data across different data sources and improve interoperability in health research.

**Objective:**

The aim of our study was to provide a systematic review of existing literature and determine the current state of FHIR implementations in health research and possible future directions.

**Methods:**

We searched the PubMed/MEDLINE, Embase, Web of Science, IEEE Xplore, and Cochrane Library databases for studies published from 2011 to 2022. Studies investigating the use of FHIR in health research were included. Articles published before 2011, abstracts, reviews, editorials, and expert opinions were excluded. We followed the PRISMA (Preferred Reporting Items for Systematic Reviews and Meta-Analyses) guidelines and registered this study with PROSPERO (CRD42021235393). Data synthesis was done in tables and figures.

**Results:**

We identified a total of 998 studies, of which 49 studies were eligible for inclusion. Of the 49 studies, most (73%, n=36) covered the domain of clinical research, whereas the remaining studies focused on public health or epidemiology (6%, n=3) or did not specify their research domain (20%, n=10). Studies used FHIR for data capture (29%, n=14), standardization of data (41%, n=20), analysis (12%, n=6), recruitment (14%, n=7), and consent management (4%, n=2). Most (55%, 27/49) of the studies had a generic approach, and 55% (12/22) of the studies focusing on specific medical specialties (infectious disease, genomics, oncology, environmental health, imaging, and pulmonary hypertension) reported their solutions to be conferrable to other use cases. Most (63%, 31/49) of the studies reported using additional data models or terminologies: Systematized Nomenclature of Medicine Clinical Terms (29%, n=14), Logical Observation Identifiers Names and Codes (37%, n=18), International Classification of Diseases 10th Revision (18%, n=9), Observational Medical Outcomes Partnership common data model (12%, n=6), and others (43%, n=21). Only 4 (8%) studies used a FHIR resource from the domain “Public Health & Research.” Limitations using FHIR included the possible change in the content of FHIR resources, safety, legal matters, and the need for a FHIR server.

**Conclusions:**

Our review found that FHIR can be implemented in health research, and the areas of application are broad and generalizable in most use cases. The implementation of international terminologies was common, and other standards such as the Observational Medical Outcomes Partnership common data model could be used as a complement to FHIR. Limitations such as the change of FHIR content, lack of FHIR implementation, safety, and legal matters need to be addressed in future releases to expand the use of FHIR and, therefore, interoperability in health research.

## Introduction

Within the current COVID-19 pandemic, there was a broad realization of the currently limited data collection processes and how powerful the exchange of scientific data could be if interoperability between health care and research was provided [1]. Although there was a large amount of data in the health care ecosystem, there was lack of data that adheres to Findable, Accessible, Interoperable, and Reusable [2] principles for users to find, use, analyze, and share data on COVID-19. This applies specifically to academic health research where the lack of interoperability between health care and research often inhibits the use of existing data sources for research. Commonly, the data collections of health research are stored in decentralized, autonomous data infrastructures which requires integration into common frameworks to enable centralized search and access.

However, processing national and cross-national scientific data across different institutions and software systems requires international standards and terminologies: the Observational Health Data Sciences and Informatics (OHDSI) Observational Medical Outcomes Partnership (OMOP) common data model (CDM) is used in observational research, whereas the Clinical Data Interchange Standards Consortium (CDISC) Operational Data Standard (ODM) is used specifically for the exchange of data within clinical trials [3]. CDISC is providing standards such as standardized raw data sets (Study Data Tabulation Model; SDTM), also considered a CDM, as well as standardized analysis data sets models. Further established standards are the terminologies Systematized Nomenclature of Medicine Clinical Terms (SNOMED CT) and Logical Observation Identifiers, Names, and Codes (LOINC). SNOMED CT is the most comprehensive clinical health care terminology worldwide providing more than 350,000 concepts, whereas LOINC is a standard for laboratory tests and clinical observations. One of the latest emerging standards for the exchange of health data is the standard Fast Healthcare Interoperability Resources (FHIR).

FHIR is a standard used in health information technology introduced in 2011 by the Standard Developing Organization Health Level Seven International (HL7). FHIR is based on previous HL7 standards (HL7 versions 2 and 3 and Clinical Document Architecture) and combines their advantages with established modern web technologies such as a Representational State Transfer (REST) architecture; application programming interface (API), XML, and JSON formats; and authorization tools (Open Authorization). In FHIR, all exchangeable content is defined by distinct basic building blocks—referred to as resources—which define the content and structure of information and can refer to each other using reference mechanisms [4].

The base FHIR specification serves as a foundation providing basic resources, frameworks, APIs, and a platform in which different solutions can be implemented [5]. To cover information not included in the basic resources, FHIR provides a built-in extension mechanism and can be adapted for specific use cases while ensuring interoperability. Additional rules and constraints within resources can be defined in profiles. Therefore, FHIR covers various domains of health care with its resources and can be used for different purposes and in various contexts and workflows.

With regard to health research, there is still a lack of use of international standards when exchanging data between health care and research institutions. However, there have been recent regulative and legislative changes promoting standards and interoperability in health care [6-8]. In addition, there are initiatives of HL7 promoting FHIR’s use in health research, such as the Vulcan HL7 FHIR Accelerator aiming to connect clinical research and health care, the MedMorph project aiming to advance public health by using standards such as FHIR, and the collaboration of HL7 and OHDSI on a single common data model [9-11]. As many research platforms and modern data management systems, such as the Extensible Neuroimaging Archive Toolkit open-source imaging informatics platform, use extensible REST APIs [12,13], FHIR may be the new standard to fill the interoperability gap in health research with its REST architecture. Existing reviews on FHIR investigate the general use of FHIR in digital health [14] or its use in electronic health records [15]. However, to the best of our knowledge, the use of FHIR in health research has not been systematically investigated. Therefore, the aim of our study was to provide a systematic review of existing literature to determine the current state of use cases, implementation, goals, and limitations of FHIR in health research.

## Methods

### Protocol, Registration, and Ethical Considerations

This systematic review was conducted in accordance with the (PRISMA) Preferred Reporting Items for Systematic Reviews and Meta-Analyses guidelines [16]. The review was registered with the International Prospective Register of Systematic Reviews (PROSPERO; CRD42021235393) [17]. As data originated from published studies, ethical approval for this study was not requested.

### Inclusion and Exclusion Criteria 

We included studies investigating the use of FHIR in health research. We did not focus on particular patient populations, interventions, control groups, or outcomes, except the use of FHIR in health research. Details on inclusion and exclusion criteria are presented in Textbox 1.

**Inclusion and exclusion criteria for paper review.**

Inclusion criteria

- Studies focusing on the use of FHIR in health care research 
- Original papers published in peer-reviewed journals in English 
- Studies with publication dates no earlier than 2011 

Exclusion criteria

- Studies focusing on the use of FHIR in electronic health records, mobile and web apps, decision support, and data protection or security
- General overviews on FHIR 
- Comments, books, editorials, or reviews 
- Language other than English
- Studies conducted before 2011 

### Information Sources and Search Strategy 

A comprehensive literature search was performed through the PubMed/MEDLINE, Embase, Web of Science, IEEE Xplore, and Cochrane Library databases. In addition, citation tracking and reference list checking were performed. The goal of the search strategy was to retrieve all relevant studies related to our research question published between 2011 and 2022. Search terms were therefore relatively broad to make sure that all potentially relevant studies were identified. Search terms used for the database searches were “FHIR” and “Fast Healthcare Interoperability Resources.” Information on the detailed search strategy for each database is provided as an appendix to this review (Multimedia Appendix 1). The search was conducted on February 26, 2022.

### Study Selection and Data Collection Process 

Study selection included 2 screening levels: (1) screening of titles and abstracts of all studies identified in the literature search and (2) full-text review of studies that had not been excluded in the first step. Review at the first stage of screening was performed independently by 2 authors (ML and SAIK) using the Rayyan web app [18]. Remaining disagreements were resolved by a third author (CNV). Further full-text screening at the second stage and data extraction were performed by 6 authors (CNV, ML, SAIK, PJM, AB, and TH), and disagreements of at least two authors at this stage were resolved by the last author (ST).

### Data Extraction and Analysis 

Data synthesis was conducted in tables and figures. For categorical variables, simple and relative frequencies and proportions were used. To identify the networks of coauthors, we also performed a network analysis that investigated, for all authors of the included studies, whether they were coauthors in a study. Results were visualized in a network graph. We did not assess bias in studies due to the lack of quantitative tools applicable to technical papers on standards. All analyses were done with R statistical software (version 4.0.5; R Foundation for Statistical Computing) [19] and the *tidyverse* packages [20]. All data and analyses scripts are provided in a GitHub repository [21].

## Results

### Study Selection and Extraction

**Figure 1:**
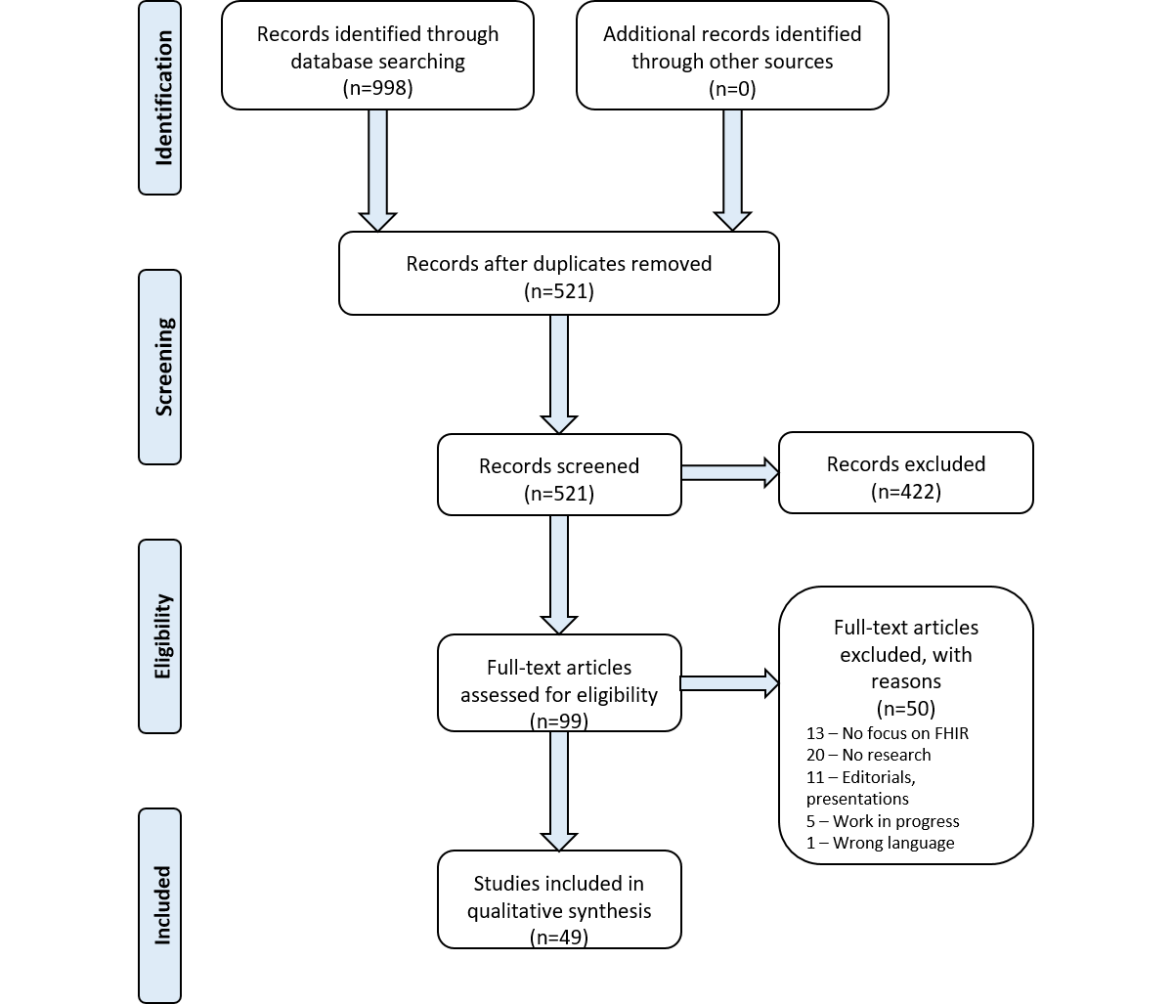
PRISMA (Preferred Reporting Items for Systematic Reviews and Meta-Analyses) flow diagram for identifying articles eligible for inclusion. FHIR: Fast Healthcare Interoperability Resources.

A total of 998 articles were identified through the database searches (344 from MEDLINE, 359 from Embase, 201 from Web of Science, 84 from IEEE Xplore, and 10 from Cochrane Library). No additional records were identified through citation tracking and reference list checking. We excluded 477 duplicates and 422 articles that did not meet the inclusion criteria or met the exclusion criteria. Among the 99 full-text articles assessed for eligibility, an additional 50 studies were excluded. Finally, 49 [1,12,22-68] articles met the inclusion criteria and were included in the systematic review (Figure 1). Details on the exclusion reasons for the full-text evaluation can be found in Multimedia Appendix 2, and the exclusion reasons for the abstract evaluation can be found in the GitHub repository [21].

### Characteristics of Included Studies

**Figure 2:**
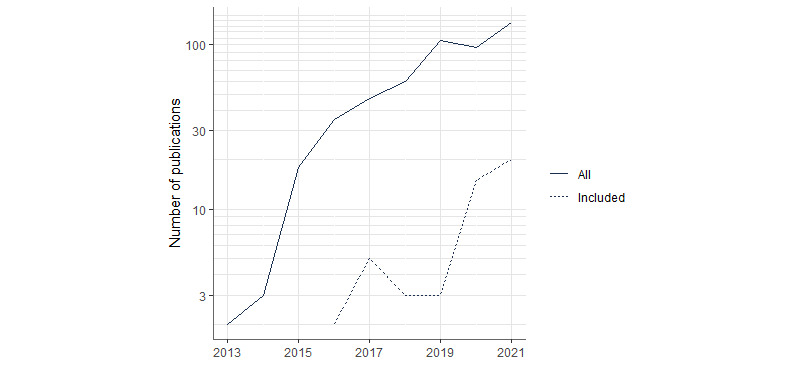
Number of publications per year (all: all FHIR publications identified in the databases with the search terms “FHIR” OR “Fast Healthcare Interoperability Resources”; included: studies included in this review). FHIR: Fast Healthcare Interoperability Resources.

Publication dates ranged from 2016 to 2022 with the median in 2020. Of the 49 included studies, 73% (n=36) were published between 2020 and 2022. The increase of publications from 2020 onward is visualized in Figure 2, showing the temporal trend of all FHIR publications identified in the databases with the search terms “FHIR” OR “Fast Healthcare Interoperability Resources” and the number of publications included into the analysis per year.

**Figure 3:**
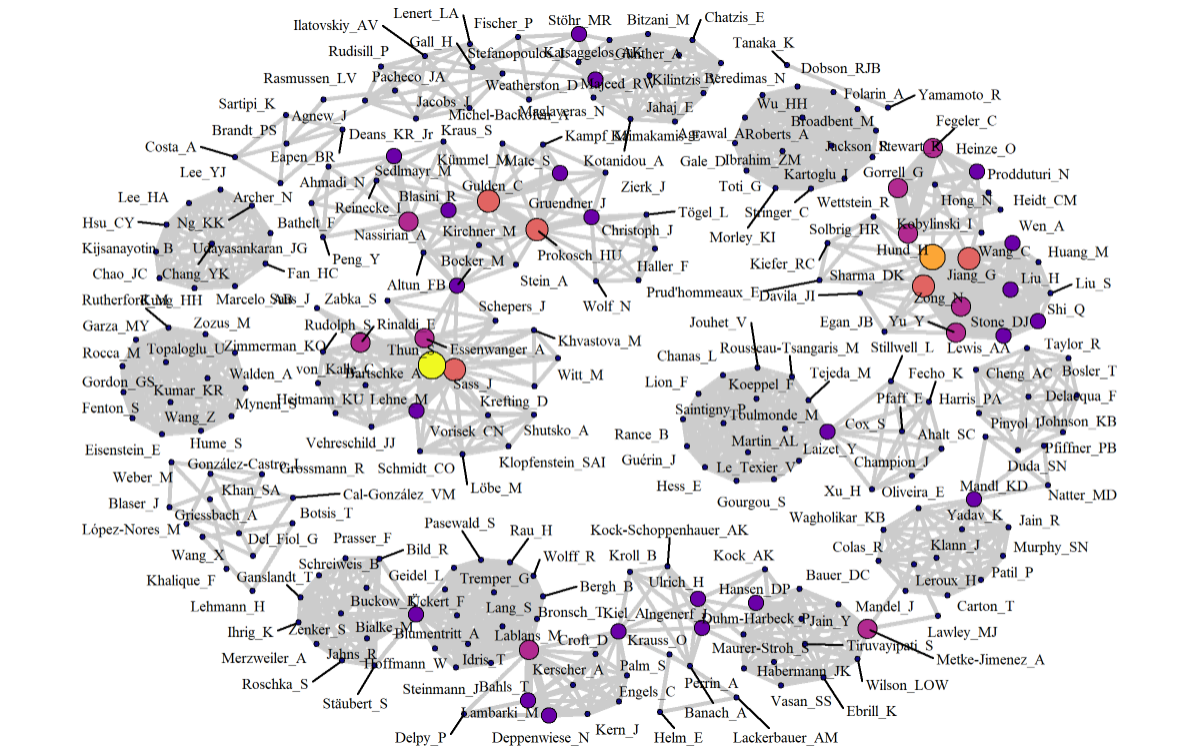
Network of coauthorships. Each point represents an author. Point size and color indicate the number of publications of this author (between 1 and 6). Lines indicate that authors have coauthored at least one paper together. Line thickness represents the number of coauthorships.

The results of the network analysis of coauthorships are shown in Figure 3. Of a total of 256 authors, most (85%, n=217) appeared only once in the included studies, and no author occurred more than 6 times within the included studies. Most coauthorship networks were restricted to individual studies, with occasional connections between networks (ie, authors having published studies with different groups of coauthors).

Of the 49 studies, the majority were conducted in Germany (47%, n=23) [12,26,28-31,34,35,40-42,45-47,52,53,56-58,60,62, 63,69], the United States (27%, n=13) [22,25,36,44,48-50,61, 64-66,68,70], and Australia (6%, n=3) [1,43,67]. The remaining studies were performed in Austria (2%, n=1) [32], Canada (2%, n=1) [24], France (2%, n=1) [51], Greece (2%, n=1) [59], Japan (2%, n=1) [27], Pakistan (2%, n=1) [38], Spain (2%, n=1) [55], Switzerland (2%, n=1) [39], Taiwan (2%, n=1) [23], and the United Kingdom (2%, n=1) [37].

### Research Domain and Area of FHIR Application

**Figure 4:**
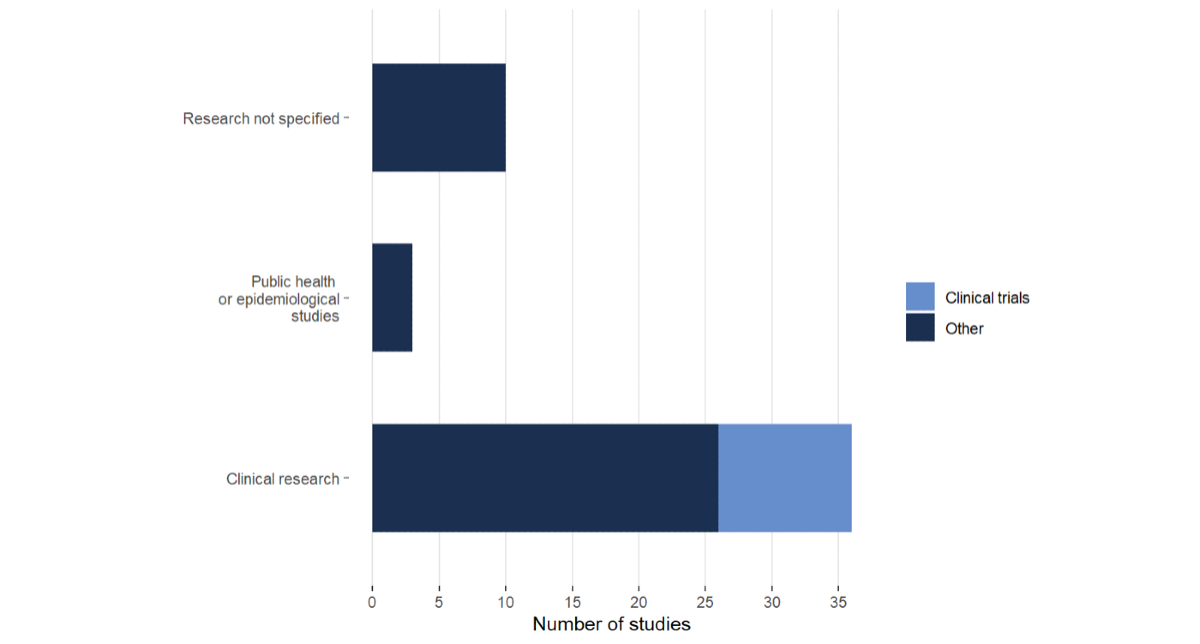
Number of studies according to research domain.

Of the 49 studies, most (73%, n=36) studies covered the research domain of clinical research, of which 10 (20%) studies were clinical trials [22,29-31,36,39,43,56,65,66]; 3 (6%) studies focused on solutions in public health and epidemiology [38,40,64], and the remaining studies did not specify their research domain (20%, n=10; Figure 4) [24,32,41,42,45-47, 50,63,69]. The included studies used FHIR for the standardization of data (41%, n=20) [23,26,30,34,41,45-48, 51-53,57-60,63,66,67,70], data capture (29%, n=14) [1,12,22,24,27,35-37,43,44,55,61,64,65], recruitment (14%, n=7) [28,29,31,32,49,56,62], analysis (12%, n=6) [25,38,42,50,68,69], and consent management (4%, n=2; Table 1) [39,40]. Details on the included studies are presented in Table 2.

**Table 1:** Numbers of studies according to area of FHIR application, medical specialty, and international standard.

Area			Studies (N=49), n (%)
**FHIR^a^ application**			
	Standardization of data	20 (41)	
	Data capture	14 (29)	
	Recruitment	7 (14)	
	Analysis	6 (12)	
	Consent management	2 (4)	
**Medical specialty**			
	Generic approach	27 (55)	
	Infectious disease	8 (16)	
	Oncology	6 (12)	
	Genomics	4 (8)	
	Pulmonary hypertension	1 (2)	
	Neuroimaging research	1 (2)	
	Genomic cancer medicine	1 (2)	
	Environmental health	1 (2)	
**International standard**			
	Other	21 (43)	
	None	18 (37)	
	LOINC^b^	14 (29)	
	SNOMED CT^c^	18 (37)	
	ICD-10^d^	9 (18)	
	OMOP^e^	6 (12)	

^a^FHIR: Fast Healthcare Interoperability Resources.

^b^LOINC: Logical Observation Identifiers Names and Codes.

^c^SNOMED CT: Systematized Nomenclature of Medicine Clinical Terms.

^d^ICD-10: International Classification of Diseases 10th Revision.

^e^OMOP: Observational Medical Outcomes Partnership.

**Table 2:** Characteristics of studies.

Source, year	Country	Item mapped to FHIR^a^	Objective for FHIR use	FHIR resources
Banach et al [56], 2021	Germany	Medical and demographic data from free-text eligibility criteria	Estimation of the number of potentially eligible patients for planning multicenter trials based on free-text criteria and using a consented data set based on FHIR	—^b^
Bauer et al [1], 2020	Australia	Questionnaire	Ontology-based standard questionnaire for linking genomic data with clinical outcomes	Questionnaire
Bialke et al [40], 2018	Germany	Modular consent templates	Support improvement for consent definition and consent documentation	Consent
Bild et al [28], 2020	Germany	Informed consent template	Cross-site interoperability layer for representing the validity of data use policies derived from signed informed consent templates and regulatory framework	Consent and Patient
Brandt et al [71], 2021	United States	Phenotype definitions from the Phenotype Knowledgebase repository	Repository of structured phenotype definitions for automation of cohort identification.	Patients, Encounter, Procedure, Medication orders, Condition, and Observation
Cheng et al [44], 2021	United States	EHR^c^ Data	Seamless data exchange between the REDCap^d^ research electronic data capture and any EHR system with a FHIR API^e^	Patient, Observation, AllergyIntolerance,MedicationOrder, and Condition
Deppenwieset al [57], 2021	Germany	Oncology data	Provide a transformation tool from oncology data XML files to FHIR for oncological data to enable clinical research	Medication, MedicationStatement, and Procedure
Eapen et al [24], 2019	Canada	Electronic form components	Management, editing, and rendering of electronic forms in the form of an open-source framework	Questionnaire and QuestionnaireResponse
Fischer et al [35], 2020	Germany	Common data set from a German pulmonary hypertension registry	Feasibility of HL7^f^ FHIR Bundle and XSLT^g^ as a generic ETL^h^ process to populate an OMOP^i^ CDM^j^	Patient, Encounter, and Observation
Garza et al [61], 2020	United States	Concomitant medications, demographics, eligibility, labs, medical history, therapeutic area–specific, procedure, encounters, vital signs, other, administrative, questionnaires, and study drug administration	Developing and implementing a systematic mapping approach for evaluating HL7 FHIR standard coverage in multicenter clinical trials.	Observation, Patient, Specimen, Encounter, Diagnostic Report, and Condition
González- Castro et al [55], 2021	Spain	Clinical patient data (from EHR) and patient-generated data	Collection and aggregation of survivorship data (use cases colon cancer and breast cancer)	Patient, Condition, Observation, MedicationStatement, Encounter, and Procedure
Gruendner et al [69], 2020	Germany	Clinical patient data	Analysis within and across institutions	—
Gruendner et al [42], 2021	Germany	Metadata	Developing a Metadata Schema based on FHIR to gather metadata on clinical, epidemiological, and public health studies; elevate data FAIRness^k^; and widen analysis possibilities across health research domains	ResearchStudy, Questionnaire, and DocumentReference
Guérin et al [51], 2021	France	Clinical and omics data in oncology	Improve and accelerate retrospective and prospective clinical and genomic data sharing in oncology	MolecularSequence and Observation
Gulden et al [31], 2018	Germany	Eligibility criteria of clinical trials	Recruitment of patients for clinical trials using eligibility criteria	Condition and Patient
Gulden et al [30], 2021	Germany	Clinical trial data	Multisite clinical trial registry	ResearchStudy
Hong et al [25], 2017	United States	Ovarian cancer data	Support of clinical statistics and analysis leveraging standardized data exchange and access services based on FHIR	Patient, Observation, Condition, and Procedure
Hund et al [53], 2021	Germany	Process data	Developing a framework to enable standardized, shared processes using Business Process Model and Notation and FHIR for arbitrary biomedical research	ActivityDefinition, Binary, Bundle, CodeSystem, Endpoint,Group, NamingSystem, Organization, Practitioner, PractitionerRole, ResearchStudy, StructureDefinition, Subscription, and Task
Jiang et al [70], 2017	United States	Clinical research data	Development and assessment of a consensus-based approach for harmonizing the OHDSI^l^ CDM with HL7 FHIR	Observation
Kilintzis et al [59], 2022	Greece	Clinical information from in-ICU^m^ COVID-19 patients	Fusion of clinical information with chest sounds and imaging of COVID-19 ICU patients	Media
Klopfenstein et al [41], 2021	Germany	Metadata of clinical, epidemiological and public health studies	Developing a Metadata Schema based on FHIR to gather metadata on clinical, epidemiological, and public health studies; elevate data FAIRness; and widen analysis possibilities across health research domains	ResearchStudy, Questionnaire, and DocumentReference
Khalique and Khan [38], 2017	Pakistan	EHR	Analysis or mining of EHR data and contextual information to assess the population’s health	—
Khvastova et al [12], 2020	Germany	Open-source research platform (XNAT^n^)	Feasibility study for the full integration of FHIR into XNAT	Patient
Lackerbauer et al [32], 2019	Austria	Informed consent or questionnaires	Automated verification of answers	Questionnaire and QuestionnaireResponse
Lambarki et al [58], 2021	Germany	Oncology data	Use and apply a harmonized FHIR-based modular data set in a federated data platform for translational cancer research to store data in a structured manner and enable data transfer	Condition, Observation, Procedure, MedicationStatement, Patient, Organization, Specimen, ClinicalImpression, Encounter, and ServiceRequest
Lee et al [23], 2020	Taiwan	IPS^o^	FHIR-based global infectious disease surveillance and case-tracking model	MedicationStatement, Medication, AllergyIntolerance, Condition, Immunization, Procedure, Organization, Observation, CarePlan, and Location
Lenert et al [50], 2021	United States	Clinical data	Availability of data for research	Patient, Encounter, Condition, Procedure, Observation, MedicationRequest, and MedicationAdministration
Leroux et al [67], 2017	Australia	Data model	Mapping CDISC^p^ ODM^q^ to FHIR	Patient, Observation, EpisodeOfCare, Encounter, QuestionnaireResponse, Questionnaire, and CarePlan
Majeed et al [60], 2021	Germany	General patient information, encounter, or visit related information; individual data points; observations; measurements; and surveys	Developing a generic ETL framework to process patient data into FHIR and enable data integration in a single central data warehouse as a prerequisite for translational research	Patient, Observation, and Encounter
Metke-Jimenez et al [43], 2019	Australia	REDCap forms	Data export from REDCap into FHIR resources	Encounter, Observation, Condition, and Patient
Peng et al [52], 2021	Germany	Genomic Variant Cell Format data	Coverage of Variant Cell Format data in OMOP CDM with and without using FHIR as intermediate layer	MolecularSeqeunce, Patient, and Condition
Pfiffner et al [22], 2016	United States	ResearchKit data	Patient-reported outcomes	Contract, Questionnaire, QuestionnaireResponse, Patient, and Observation
Reinecke et al [29], 2020	Germany	Patient ID lists	Data-driven recruitment of patients for clinical trials, storage of patient lists, and generation of notifications	List
Rinaldi et al [45], 2021	Germany	Microbiology data	Standardization of clinical data from patient care and medical research in the field of infection control	DiagnosticReport, Observation, Specimen, and ServiceRequest
Rinaldi et al [47], 2021	Germany	OpenEHR Template	Mapping infection control related data across 3 different standards—OpenEHR, FHIR, and OMOP CDM—to maximize analysis capabilities	DiagnosticReport, Observation, Specimen, ServiceRequest, and Encounter
Sass et al [26], 2020	Germany	COVID-19 data	Standardized data model	Patient, Consent, Observation, Condition, Procedure, Encounter, Medication, and MedicationStatement
Sass et al [46], 2021	Germany	Medication chapter of the German Procedure Classification and Identification of Medicinal Products–compliant medication terminology	Representation of structured medication data	Patient, Procedure, MedicationStatement, and Medication
Tanaka et al [27], 2020	Japan	SS-MIX2^r^	Mapping electronic medical record items between SS-MIX2 and HL7 FHIR	Patient, Encounter, Condition, AllergyIntolerance, Observation, Specimen, ServiceRequest, MedicationRequest, and MedicationDispense
Ulrich et al [34], 2016	Germany	Metadata or CRF^s^	Metadata repository	Questionnaire
Wagholikar et al [36], 2017	United States	Common data model demographics, laboratory results, and diagnoses	Clinical apps sharing via a platform	—
Wang et al [48], 2021	United States	FDA^t^’s Adverse Event Reporting System data	Potential use of FHIR for postmarket safety surveillance for drug products	AdverseEvent
Weber et al [39], 2020	Switzerland	Electronic consent form	Designing of a FHIR-based eConsent app for Android and evaluation of acceptance	Contract
Wettstein et al [62], 2021	Germany	Clinical data	Using FHIR for automated and distributed feasibility queries to find available cohort sizes across institutions	Group, ResearchStudy, and Task
Wettstein et al [63], 2021	Germany	Medical routine data	HL7 FHIR version R4 is used to define the necessary communication messages as well as process input and output variables.	Group, ResearchStudy, and Task
Wu et al [37], 2018	United Kingdom	EHR data and unstructured documents	Semantic search system for obtaining clinical insights from unstructured clinical notes	Patient and DocumentReference
Xu et al [64], 2020	United States	Data set of patients with “asthma-like” conditions	Impact of airborne pollutant exposures on asthma (research question)	—
Zong et al [65], 2020	United States	Colorectal cancer report	Automatic population of eCRFs in colorectal clinical cancer trials	Questionnaire and QuestionnaireResponse
Zong et al [66], 2021	United States	Colorectal cancer data model	Framework for capturing common data elements from CRFs and FHIR resources to identify clinical information needs	DiagnosticReport and Observation
Zong et al [68], 2020	United States	EHR	Discovery of genotype-phenotype associations	Condition, and Observation

^a^FHIR: Fast Healthcare Interoperability Resources.

^b^Not available.

^c^EHR: electronic health record.

^d^REDCap: Research Electronic Data Capture.

^e^API: application programming interface.

^f^HL7: Health Level Seven International.

^g^XSLT: Extensible Stylesheet Language Transformations.

^h^ETL: Extract-Transform-Load.

^i^OMOP: Observational Medical Outcomes Partnership.

^j^CDM: common data model.

^k^FAIR: Findable, Accessible, Interoperable, and Reusable.

^l^OHDSI: Observational Health Data Sciences and Informatics

^m^ICU: intensive care unit.

^n^XNAT: Extensible Neuroimaging Archive Toolkit.

^o^IPS: International Patient Summary.

^p^CDISC: Clinical Data Interchange Standards Consortium.

^q^ODM: Operational Data Model.

^r^SS-MIX2: Standardized Structured Medical Information Exchange2.

^s^CRF: Case Report Form.

^t^FDA: U.S. Food and Drug Administration.

### Study Objectives

In terms of medical specialty, most (55%, 27/49) of the studies [24,27-32,34,36-42,44,46,48,49,53,56,60-63,67,70] were using a generic approach—implementable in any kind of specialty (Table 2). Of the remaining studies, 16% (8/49) use cases focused on infectious disease [1,22,23,26,45,47,50,59], whereas 12% (6/49) focused on oncology [25,55,57,58,65,66] and 8% (4/49) on genomics [43,52,68,69]. Further medical specialties were environmental health (2%, 1/49) [64], genomic cancer medicine (2%, 1/49) [51], neuroimaging research (2%, 1/49) [12], and pulmonary hypertension (2%, 1/49) [35]. Despite studies implementing FHIR in specific use cases, 55% (12/22) of the studies [1,12,22,23,25,35,43,50,52,58,64,69] reported generic solutions conferrable to other use cases. Details on study objectives with regards to FHIR use can be found in Table 2 and Multimedia Appendix 3.

### International Standards

Among the 49 studies, 37% (n=18) did not report on or use additional standards or terminologies [12,22-24,27,28,30-32, 38,39,48,50,55,57,64,66,69]. SNOMED CT [1,25,26,35,37,43, 45-47,51,55,56,65,70] and LOINC [25,26,35,37,42-45, 47,49,51,55,56,58,61,65,68,70] were reported to be used by 29% (n=14) and 37% (n=18) of the studies, respectively; 18% (n=9) of the studies used International Classification of Diseases 10th Revision [25,26,35,37,49,51,58,65,68] and 12% (n=6) used OMOP CDM [26,29,35,47,52,60]; and 43% (n=21) of the studies used additional standards which were categorized under “Other” (Table 1) [26,34-37,40,42,43,45-47,49,51,56,58-60, 62,63,67,70]. The implemented FHIR resources by each study are listed in Table 2; 5 (10%) studies did not precisely list their FHIR resources used [36,38,56,64,69]. Information on the FHIR version used was provided by 45% (n=22) of the studies [22,23,25,26,28,30,32,35,40,42,48,49,57,59,60,66,68,70], which can be found in Multimedia Appendix 4.

### Limitations of FHIR Use

With regard to the limitations of FHIR use, Bild et al [28], Lackerbauer et al [32], and Metke-Jimenez et al [43] reported the possible content changes of new versions of FHIR resources. Generalizability was a concern in the studies of Khalique et al [38] and Zong et al [65]. The need for a FHIR server [69] and the requirement for a protocol for deidentification [1] were additional limitations. Reinecke et al [29] had not tested the exchange of data between locations and therefore could not provide information in terms of use and results of their prototype. Wagholikar et al [36] implemented a limited subset of FHIR resources in their platform and therefore the filtering of FHIR resources using complex query formats was not supported. In terms of electronic consents, safety and legal matters were major concerns [39]. Zong et al [68], investigating the discovery of genotype-phenotype associations, reported the lack of information on differences in genetic data as well as extra mapping efforts since the data were from multiple sources. In addition, there was a lack of resources preventing the demonstration of use in the study. Generalizability was also a concern in this study in terms of exploring the FHIR framework within other variants and noncancer phenotypes in future work.

## Discussion

### Principal Findings

This systematic review summarizes the current state of use cases implementing FHIR in health research. As FHIR was developed in 2011, we included studies from 2011 to 2022 and found that half of studies were published between 2020 and 2022, displaying an increased use of FHIR in the past years. Interestingly, the first publication of our included studies emerged in 2016, indicating a 5-year latency between the publication of the FHIR standard and the publication of studies addressing its use in health research. Germany and the United States were the countries with the highest number of publications, which might be due to recent regulatory measurements and initiatives: in the United States, the 21st Century Cures Act requires the use of FHIR for health data; and in Germany, the medical informatics initiative aiming to close the gap between research and health care used FHIR in their already established use cases. Our network analysis showed that authorships were dispersed relatively equally across studies, not dominated by individual research groups or authors.

Most studies aimed to primarily standardize their data for health research and reported using additional international standards and terminologies. Within studies using FHIR for data capture, the FHIR resource “Questionnaire” was often used. Further areas of FHIR use were analysis, recruitment, and consent management. The literature shows that fast and efficient patient screening for clinical trial recruitment support systems is important, and there is a current lack of standards and interoperability of in these systems, as well as with regard to eligibility criteria [72].

The majority of studies followed a more generic approach rather than implementing FHIR for a specific use case. The studies establishing use cases focused on infectious diseases, specifically COVID-19, as well as genomics, oncology, and imaging—which are all specialties more advanced in terms of digitalization. Among these use cases, only a small number of studies reported limited generalizability of their results.

Though provided by FHIR specifically for research, resources out of the domains “Public Health & Research” and “Evidence-Based Medicine” were used in only 4 studies. A recently published study investigated the feasibility of the FHIR resource “ResearchStudy” in a metadata registry for COVID-19 research and found that there was a need for the use of extensions on more than 20% of the data items [41]. However, the resources “ResearchStudy” and “ResearchSubject” are currently under revision and will likely be tailored more to researchers’ needs when released with FHIR version R5 in 2022 [73].

Our analysis found that FHIR was used as a complement to other standards. Studies reporting on terminologies mostly used SNOMED CT and LOINC, both terminologies supported by FHIR within its value sets. There were 6 studies that used FHIR in addition to OMOP CDM, a standard widely used in observational research. Using OMOP CDM, a recommended way of transforming and transferring data from existing databases—Extract-Transform-Load tools are used for each source separately. To connect multiple heterogeneous databases, FHIR can be used as an intermediate format for local data extraction [35]. Reinecke et al [29] also extended the OMOP CDM with FHIR to exchange electronic health record data to connect the CDM to several health care systems. However, there were also limitations as Leroux et al [67] mapped CDISC SDTM and FHIR and found that CDISC SDTM’s use of controlled terminology is inhibiting semantic interoperability solutions; FHIR uses semantic standards accepted in health care that are usually precoordinated (eg, SNOMED CT and LOINC), whereas CDISC SDTM uses only controlled terminology in postcoordination. Therefore, there would be the need for sponsors to translate terminologies used within systems. Leroux et al [67] proposed the new FHIR resources “ClinicalStudyPlan” and “ClinicalStudyData”—equivalent to ODM “Study” and “ClinicalData” elements—which could overcome the semantic incompatibility. However, mappings with data transformation may lead to information loss and errors; therefore, developing ODM toward FHIR would be preferable, and the draft of ODM version 2.0 already includes better support for FHIR [74,75]. In addition, HL7 and CDISC have jointly released a mapping implementation guide to help transform FHIR content into CDISC Clinical Data Acquisition Standards Harmonization Implementation Guide or SDTM Implementation Guide data sets. [76,77].

With regard to limitations using FHIR, there were certain drawbacks reported by the included studies such as the possible change in the content of different versions of FHIR resources, safety, legal matters, and the need for a FHIR server. Not all studies tested the use of FHIR in practice and, therefore, could not provide results on the actual FHIR implementation.

### Limitations

One limitation of our study is the lack of quality evaluation due to missing established tools for evaluating technical papers on standardization in health care. For technical evaluations, structured information on additional standards, software, and FHIR version was missing in several studies. Therefore, our analysis on additional used standards might be biased as half of the studies did not report on using other international standards or terminologies. In addition, there were studies that did not list their FHIR resources clearly or at all. We aimed to guarantee an optimal systematic review process targeting academic peer-reviewed literature that is available in English; however, limitations remained as we may have missed relevant studies that were not published in the target language. Furthermore, we assumed that the published literature provides a surplus on successful FHIR initiatives because, in general, unsuccessful initiatives tend to stay unpublished [78]. Thus, our review may suffer from publication bias. In addition, this study investigated studies with a clear focus on FHIR in health research. However, there might be research projects using FHIR without FHIR being the central message or included in title and abstract.

### Conclusions

To the best of our knowledge, this is the first systematic review investigating the use of FHIR in health research. It was shown that FHIR has been successfully implemented in clinical, public health, and epidemiological research at the stages of recruitment and consent management, data capture, and standardization as well as analysis of patient data. The implementation of international terminologies such as SNOMED CT and LOINC is common and, together with the REST API, stands out in comparison with other health research standards. Other standards such as OMOP CDM were used as a complement to FHIR in some studies, and a future aim could be the development of an infrastructure for the seamless integration and communication of health information across different standards. This approach is reinforced by the current development of collaborations of different Standards Developing Organizations such as OHDSI and FHIR and the improved support of FHIR in combination with CDISC. Resources of the domain “Public Health & Research” and “Evidence-Based Medicine” were rarely used and could further elevate interoperability in health research, specifically after their modifications in FHIR version R5. However, this approach will need to address current limitations but could, if successfully implemented, elevate digitalized health research.

## Appendix

Multimedia Appendix 1 Search strategy for each database.

Multimedia Appendix 2 Reasons for the exclusion of full-text evaluation.

Multimedia Appendix 3 Word cloud showing the keywords of the main FHIR use objectives of the studies. FHIR: Fast Healthcare Interoperability Resources.

Multimedia Appendix 4 Additional information on the included studies.

## Abbreviations

API: application programming interface
CDISC: Clinical Data Interchange Standards Consortium
CDM: common data model
FHIR: Fast Healthcare Interoperability Resources
HL7: Health Level Seven International
LOINC: Logical Observation Identifiers Names and Codes
ODM: Operational Data Standard
OHDSI: Observational Health Data Sciences and Informatics
OMOP: Observational Medical Outcomes Partnership
PRISMA: Preferred Reporting Items for Systematic Reviews and Meta-Analyses
PROSPERO: International Prospective Register of Systematic Reviews
REST: Representational State Transfer
SDTM: Study Data Tabulation Model
SNOMED CT: Systematized Nomenclature of Medicine Clinical Terms
