# Supplementary material for: Fast Healthcare Interoperability Resources (FHIR) for Interoperability in Health Research: Systematic Review
Source: JMIR Med Inform. 2022 Jul 19;10(7):e35724. doi: 10.2196/35724 (PMC9346559; doi:10.2196/35724)
Supplement: Multimedia Appendix 1 [file medinform_v10i7e35724_app1.docx]

**Search Conducted on Feb 26^th^ 2022**

**Addition File 1.** Literature sources and keywords

| **Literature sources** | **Search query** | **Return value** |
| --- | --- | --- |
| Pubmed/Medline | (((("FHIR"[Title/Abstract]) OR ("Fast Healthcare Interoperability Resources"[Title/Abstract]))) AND (("2011"[Date - Publication] : "2022/02/26"[Date - Publication]))) AND (english[Language]) | 344 |
| Embase | (fhir:ab,ti OR 'fast healthcare interoperability resources':ab,ti) AND [2011-2022]/py0 | 359 |
| Web of Science | (TS=(FHIR)) OR TS=(Fast healthcare interoperability resources) Timespan: 2010-01-01 to 2022-02-26 (Publication Date) | 201 |
| IEEE Xplore | "Document Title":"FHIR" OR "Document Title":"Fast Healthcare Interoperability Resources" OR "Abstract":"FHIR" OR "Abstract":"Fast Healthcare Interoperability Resources"  + Filter (2011-2021)* | 84 |
| Cochrane | (FHIR):ti,ab,kw OR (Fast Healthcare Interoperability Resources):ti,ab,kw (Word variations have been searched)" with Cochrane Library publication date Between Jan 2011 and Feb 2022, in Cochrane Reviews, Trials (Word variations have been searched) | 10 |

*Not possible to select 2022 in IEEE Xplore
