# Supplementary material for: Fast Healthcare Interoperability Resources (FHIR) for Interoperability in Health Research: Systematic Review
Source: JMIR Med Inform. 2022 Jul 19;10(7):e35724. doi: 10.2196/35724 (PMC9346559; doi:10.2196/35724)
Supplement: Multimedia Appendix 2 [file medinform_v10i7e35724_app2.docx]

| **Title** | **Authors** | **Reason for exclusion** |
| --- | --- | --- |
| Fast Healthcare Interoperability Resources (FHIR) as a Meta Model to Integrate Common Data Models: Development of a Tool and Quantitative Validation Study. | Pfaff ER, Champion J, Bradford RL, Clark M, Xu H, Fecho K, Krishnamurthy A, Cox S, Chute CG, Overby Taylor C, Ahalt S | No focus on research |
| Sync for Genes: Making Clinical Genomics Available for Precision Medicine at the Point-of-Care. | Garcia SJ, Zayas-Cabán T, Freimuth RR | No focus on research |
| Profiling Fast Healthcare Interoperability Resources (FHIR) of Family Health History based on the Clinical Element Models. | Lee J, Hulse NC, Wood GM, Oniki TA, Huff SM | No focus on research |
| KETOS: Clinical decision support and machine learning as a service - A training and deployment platform based on Docker, OMOP-CDM, and FHIR Web Services. | Gruendner J, Schwachhofer T, Sippl P, Wolf N, Erpenbeck M, Gulden C, Kapsner LA, Zierk J, Mate S, Stürzl M, Croner R, Prokosch HU, Toddenroth D | No focus on FHIR |
| QL(4)MDR: a GraphQL query language for ISO 11179-based metadata repositories. | Ulrich H, Kern J, Tas D, Kock-Schoppenhauer AK, Ückert F, Ingenerf J, Lablans M | No focus on FHIR |
| Primary care perspectives on implementation of clinical trial recruitment. | Taft T, Weir C, Kramer H, Facelli JC | No focus on FHIR |
| MDRCupid: A Configurable Metadata Matching Toolbox. | Deppenwiese N, Duhm-Harbeck P, Ingenerf J, Ulrich H | No focus on research |
| Improving Interoperability between Registries and EHRs. | Blumenthal S | Work in progress |
| Public health reporting and outbreak response: synergies with evolving clinical standards for interoperability. | Mishra NK, Duke J, Lenert L, Karki S | No focus on research |
| From Raw Data to FAIR Data: The FAIRification Workflow for Health Research. | Sinaci AA, Núñez-Benjumea FJ, Gencturk M, Jauer ML, Deserno T, Chronaki C, Cangioli G, Cavero-Barca C, Rodríguez-Pérez JM, Pérez-Pérez MM, Laleci Erturkmen GB, Hernández-Pérez T, Méndez-Rodríguez E, Parra-Calderón CL | Work in progress |
| Improving Cancer Data Interoperability: The Promise of the Minimal Common Oncology Data Elements (mCODE) Initiative. | Osterman TJ, Terry M, Miller RS | No focus on research |
| Integrating Heterogeneous Data Sources for Cross-Institutional Data Sharing: Requirements Elicitation and Management in SMITH. | Tahar K, Müller C, Dürschmid A, Haferkamp S, Saleh K, Jürs P, Stäubert S, Gewehr JE, Zenker S, Ammon D, Wendt T | No focus on FHIR |
| HiGHmed - An Open Platform Approach to Enhance Care and Research across Institutional Boundaries. | Haarbrandt B, Schreiweis B, Rey S, Sax U, Scheithauer S, Rienhoff O, Knaup-Gregori P, Bavendiek U, Dieterich C, Brors B, Kraus I, Thoms CM, JÃ¤ger D, Ellenrieder V, Bergh B, Yahyapour R, Eils R, Consortium H, Marschollek M | No focus on FHIR |
| The Pluripotent Rendering of Clinical Data for Precision Medicine. | Chute CG, Huff SM | No focus on FHIR |
| Reliability, Feasibility, and Patient Acceptance of an Electronic Version of a Multidimensional Health Assessment Questionnaire for Routine Rheumatology Care: Validation and Patient Preference Study. | Pincus T, Castrejon I, Riad M, Obreja E, Lewis C, Krogh NS | No focus on FHIR |
| Data Integration for Future Medicine (DIFUTURE). | Prasser F, Kohlbacher O, Mansmann U, Bauer B, Kuhn KA | Work in progress |
| Towards a Single Data Exchange Standard for Use in Healthcare and in Clinical Research. | Aerts J | Wrong publication type |
| The Portal of Medical Data Models: Where Have We Been and Where Are We Going? | Geßner S, Neuhaus P, Varghese J, Bruland P, Meidt A, Soto-Rey I, Storck M, Doods J, Dugas M | No focus on FHIR |
| Making Science Computable: Developing Code Systems for Statistics, Study Design, and Risk of Bias. | Alper BS, Dehnbostel J, Afzal M, Subbian V, Soares A, Kunnamo I, Shahin K, McClure RC | No focus on FHIR |
| A Novel Data Aggregation Platform (DACAP) to Characterize the IBD Patient Population and Treatment Choices in a Large GI Private Practice | Korman L, Maldonado JE, Fourment C, Weinstein M, Ali A, Sahu R | Wrong publication type |
| Achieving evidence interoperability in the computer age: Setting evidence on FHIR | Alper B, Mayer M, Shahin K, Richardson J, Schilling L, Tristan M, Salas N | Wrong publication type |
| Gabriella miller kids first data resource center: Harmonizing clinical and genomic data to support childhood cancer and structural birth defect research | Heath AP, Taylor DM, Zhu Y, Raman P, Lilly J, Storm P, Waanders AJ, Ferret V, Yung C, Mattioni M, Davis-Dusenbery B, Flamig ZL, Grossman R, Volchenboum SL, Mueller S, Nazarian J, Vasilevsky N, Haendel MA, Resnick A | Wrong publication type |
| Using medical informatics to improve clinical trial operations | Eisenstein E, Nordo A, Zozus M, Adams S | No focus on FHIR |
| Establishment of technical specifications for data sharing in esophageal cancer screening cohort based on FHIR model and CDASH model | H.-X. Yue, L.-T. Gui, Y.-L. Zhan, Liu X, Zhang Y.-F., Bian F., Jiang Y | wrong language |
| Establishing the common oncology data model for the national biobank consortium of Taiwan | Yi-Hsin Y, Chen L-T, Huang S-F | wrong publication type |
| Transplant data platform-an augmented clinical intelligence framework | Focht C, Tian W, Zeng J, Dzebisashvili N, Ghosh S | wrong publication type |
| Closing the gap: How to record valuable data for movement disorders using FHIR | J., Mecklenburg and G., Wenzel and A., Kreichgauer and C., Friedow, Kuhn A A | wrong publication type |
| Advanced precision health resources in the Susan G Komen tissue bank at the IU simon comprehensive cancer center | S., Corcoran and J., Russell and J., Clayton and A., Smith and K., Keenan and M., Robson, Iyengar N | wrong publication type |
| StayHome: A FHIR-Native Mobile COVID-19 Symptom Tracker and Public Health Reporting Tool. | Burkhardt HA, Brandt PS, Lee JR, Karras SW, Bugni PF, Cvitkovic I, Chen AY, Lober WB | No focus on research |
| openEHR-to-FHIR: Converting openEHR Compositions to Fast Healthcare Interoperability Resources (FHIR) for the German Corona Consensus Dataset (GECCO). | Ladas N, Franz S, Haarbrandt B, Sommer KK, Kohler S, Ballout S, Fiebeck J, Marschollek M, Gietzelt M | No focus on research |
| Using the Electronic Health Record to Enhance Care in Pediatric Rheumatology. | Eisenstein E, Nordo A, Zozus M, Adams S | wrong publication type |
| Next Generation of Central Cancer Registries. | Wormeli P, Mazreku J, Pine J, Damesyn M | work in progress |
| Accessing the ECG Data of the Apple Watch and Accomplishing Interoperability Through FHIR., | Bartschke A, Börner Y, Thun S | No focus on research |
| Blueprint for aligned data exchange for research and public health. | Michaels M, Syed S, Lober WB | work in progress |
| Enabling Research and Clinical Use of Patient-Generated Health Data (the mindLAMP Platform): Digital Phenotyping Study. | Vaidyam A, Halamka J, Torous J | No focus on research |
| Evaluating Site-Level Implementations of the HL7 FHIR Standard to Support eSource Data Exchange in Clinical Research. | Garza MY, Rutherford MW, Adagarla B, Eisenstein E, Kumar KR, Zimmerman KO, Topaloglu U, Zozus M | No focus on research |
| Development and Validation of the Radiology Common Data Model (R-CDM) for the International Standardization of Medical Imaging Data. | Park C, You SC, Jeon H, Jeong CW, Choi JW, Park RW | No focus on FHIR |
| Mapping of OpenEHR Archetypes to FHIR Resources in Use Case Oncology. | Rajput AM, Brakollari I | No focus on research |
| FhirSpark - Implementing a Mediation Layer to Bring FHIR to the cBioPortal for Cancer Genomics. | Reimer N, Unberath P, Busch H, Ingenerf J | No focus on research |
| ASH Research Collaborative: a real-world data infrastructure to support real-world evidence development and learning healthcare systems in hematology. | Wood WA, Marks P, Plovnick RM, Hewitt K, Neuberg DS, Walters S, Dolan BK, Tucker EA, Abrams CS, Thompson AA, Anderson KC, Kluetz P, Farrell A, Rivera D, Gertzog M, Pappas G | No focus on FHIR |
| Utilising the FOXS Stack for FAIR Architected Data Access. | Meredith J, Whitehead N, Dacey M | No focus on FHIR |
| Leveraging Genetic Reports and Electronic Health Records for the Prediction of Primary Cancers: Algorithm Development and Validation Study. | Zong N, Ngo V, Stone DJ, Wen A, Zhao Y, Yu Y, Liu S, Huang M, Wang C, Jiang G | No focus on research |
| FhirExtinguisher: A FHIR Resource Flattening Tool Using FHIRPath. | Oehm J, Storck M, Fechner M, Brix TJ, Yildirim K, Dugas M | No focus on research |
| Comparing SDTM and FHIR® for Real World Data from Electronic Health Records for Clinical Trial Submissions. | Riepenhausen S, Mertens C, Dugas M | No focus on research |
| Patient-led data sharing for clinical bioinformatics research: USCDI and beyond. | Gordon WJ, Gottlieb D, Kreda D, Mandel JC, Mandl KD, Kohane IS | wrong publication type |
| A Modified Public Health Automated Case Event Reporting Platform for Enhancing Electronic Laboratory Reports With Clinical Data: Design and Implementation Study. | Mishra N and Duke J and Karki S and Choi M and Riley M and Ilatovskiy AV, Gorges M, Lenert L | No focus on research |
| Development of a FHIR Layer on Top of the OMOP Common Data Model for the CAPABLE Project. | Gabetta M, Alloni A, Polce F, Lanzola G, Parimbelli E, Barbarini N | No focus on research |
| Automated Cancer Risk Scoring Using FHIR Genomics Profiles and Secure Web Services | Harney M, Wood GM, Atnoor D, Crockett DK | wrong publication type |
| Informatics for public health and health system collaboration: Applications for the control of the current COVID-19 pandemic and the next one | Lenert LA, Ding W, Jacobs J | No focus on research |
| Quality of Life as an Indicator for Care Delivery in Clinical Oncology Using FHIR. | Beutter CNL, Ross J, Werner P, Vladimirova D, Martens UM, Fegeler C | No focus on research |
