# Supplementary figures and images for: Fast Healthcare Interoperability Resources (FHIR) for Interoperability in Health Research: Systematic Review
Source: JMIR Med Inform. 2022 Jul 19;10(7):e35724. doi: 10.2196/35724 (PMC9346559; doi:10.2196/35724)

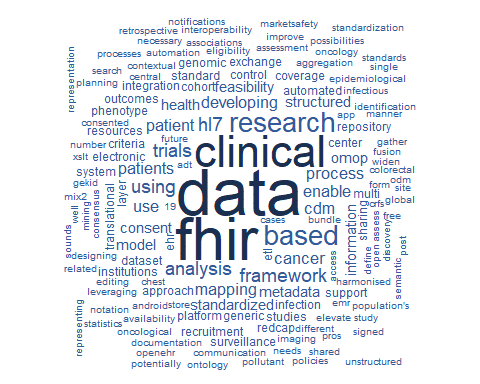

Supplement: Multimedia Appendix 3 [file medinform_v10i7e35724_app3.png]
