# Supplementary material for: Fast Healthcare Interoperability Resources (FHIR) for Interoperability in Health Research: Systematic Review
Source: JMIR Med Inform. 2022 Jul 19;10(7):e35724. doi: 10.2196/35724 (PMC9346559; doi:10.2196/35724)
Supplement: Multimedia Appendix 4 [file medinform_v10i7e35724_app4.docx]

| Source | Title | FHIR version | Area of FHIR application | Medical Area | Research Area | Clinical Trials | Journal | Impact Factor Research) | generic | Other standards - SNOMED CT | Other standards - LOINC | Other standards - ICD 10 | Other standards - OMOP | Other standards - Other | Other standards - None |
| --- | --- | --- | --- | --- | --- | --- | --- | --- | --- | --- | --- | --- | --- | --- | --- |
| Banach et al. 2021 | APERITIF - Automatic Patient Recruiting for Clinical Trials Based on HL7 FHIR. | R4 | Recruitment | Generic | Clinical Trials | 1 | Studies in Health Technology and Informatics | 0,678 | Yes | 1 | 1 | 0 | 0 | 1 | 0 |
| Bauer et al. 2020 | Interoperable medical data: The missing link for understanding COVID-19 | / | Data capture | Infectious Disease | Clinical Research | 0 | Transboundary and emerging diseases | 3,47 | Yes | 1 | 0 | 0 | 0 | 0 | 0 |
| Bialke et al. 2018 | MAGIC: once upon a time in consent management—a FHIR® tale | R4 | Consent management | Generic | Public Health/ Epidemiological Studies | 0 | Journal of Translational Medicine | 4,20 | Yes | 0 | 0 | 0 | 0 | 1 | 0 |
| Bild et al. 2020 | Towards a comprehensive and interoperable representation of consentbased data usage permissions in the German medical informatics initiative | FHIR Release 4 (v4.0.0) | Recruitment | Generic | Clinical Research | 0 | BMC medical informatics and decision making | 2,317 | Yes | 0 | 0 | 0 | 0 | 0 | 1 |
| Brandt et al. 2021 | Development of a repository of computable phenotype definitions using the clinical quality language. | / | Recruitment | Generic | Clinical Research | 1 | JAMIA Open | None | Yes | 0 | 1 | 1 | 0 | 1 | 0 |
| Cheng et al. 2021 | REDCap on FHIR: Clinical Data Interoperability Services | R4 | Data capture | Generic | Clinical Research | 0 | Journal of Biomedical Informatics | 6.317 | Yes | 0 | 1 | 0 | 0 | 0 | 0 |
| Deppenwieset al. 2021 | ADT2FHIR - A Tool for Converting ADT/GEKID Oncology Data to HL7 FHIR Resources. | / | Standardization of data | Oncology | Clinical Research | 0 | Studies in Health Technology and Informatics | 0,678 | No | 0 | 0 | 0 | 0 | 0 | 1 |
| Eapen et al. 2019 | FHIRForm: An Open-source Framework for the Management of Electronic Forms in Healthcare | / | Data capture | Generic | Research not specified | 0 | Studies in Health technology and informatics | 0.68 | Yes | 0 | 0 | 0 | 0 | 0 | 1 |
| Fischer et al. 2020 | Data Integration into OMOP CDM for Heterogeneous Clinical Data Collections via HL7 FHIR Bundles and XSLT. | FHIR standard 4.1.0 | Data capture | Pulmonary Hypertension | Clinical Research | 0 | Studies in health technology and informatics | 0.68 | Yes | 1 | 1 | 1 | 1 | 1 | 0 |
| Garza et al. 2020 | Evaluating the Coverage of the HL7 (®) FHIR (®) Standard to Support eSource Data Exchange Implementations for use in Multi-Site Clinical Research Studies. | R4 | Data capture | Generic | Clinical Research | 1 | AMIA Annual Symposium Proceedings | 1,28 | Yes | 0 | 1 | 0 | 0 | 0 | 0 |
| González- Castro et al. 2021 | CASIDE: A data model for interoperable cancer survivorship information based on FHIR. | R4 | Data capture | Oncology | Clinical Research | 0 | Journal of biomedical informatics | 6,317 | No | 1 | 1 | 0 | 0 | 0 | 1 |
| Gruendner et al. 2020 | Integrating Genomics and Clinical Data for Statistical Analysis by Using GEnome MINIng (GEMINI) and Fast Healthcare Interoperability Resources (FHIR): System Design and Implementation | / | Analysis | Genomics | Research not specified | 0 | Journal of medical Internet research | 5,03 | Yes | 0 | 0 | 0 | 0 | 0 | 1 |
| Gruendner et al. 2021 | A Framework for Criteria-Based Selection and Processing of Fast Healthcare Interoperability Resources (FHIR) Data for Statistical Analysis: Design and Implementation Study. | / | Analysis | Generic | Research not specified | 0 | JMIR MEDICAL INFORMATICS | 2,96 | Yes | 0 | 1 | 0 | 0 | 1 | 0 |
| Guérin et al. 2021 | OSIRIS: A Minimum Data Set for Data Sharing and Interoperability in Oncology. | R4 | Standardization of data | Genomic cancer medicine | Clinical Research | 0 | JCO Clin Cancer Inform | 2,95 | No | 1 | 1 | 1 | 0 | 1 | 0 |
| Gulden et al. 2018 | Investigating the Capabilities of FHIR Search for Clinical Trial Phenotyping | / | Recruitment | Generic | Clinical trials | 1 | Studies in health technology and informatics | 0.68 | Yes | 0 | 0 | 0 | 0 | 0 | 1 |
| Gulden et al. 2021 | Prototypical Clinical Trial Registry Based on Fast Healthcare Interoperability Resources (FHIR): Design and Implementation Study | R4 | Standardization of data | Generic | Clinical Trials | 1 | JMIR medical informatics | 2,58 | Yes | 0 | 0 | 0 | 0 | 0 | 1 |
| Hong et al. 2017 | Shiny FHIR: An Integrated Framework Leveraging Shiny R and HL7 FHIR to Empower Standards-Based Clinical Data Applications. | DSTU 2 | Analysis | Oncology | Clinical Research | 0 | Studies in health technology and informatics | 0.68 | Yes | 1 | 1 | 1 | 0 | 0 | 0 |
| Hund et al. 2021 | Executing Distributed Healthcare and Research Processes - The HiGHmed Data Sharing Framework. | FHIR Version 4.0.1 | Standardization of data | Generic | Clinical Research | 0 | Studies in Health Technology and Informatics | 0,678 | Yes | 0 | 0 | 0 | 0 | 0 | 0 |
| Jiang et al. 2017 | A Consensus-Based Approach for Harmonizing the OHDSI Common Data Model with HL7 FHIR. | DSTU 2 | Standardization of data | Generic | Clinical Research | 0 | Studies in health technology and informatics | 0.68 | Yes | 1 | 1 | 0 | 0 | 1 | 0 |
| Khalique et Khan 2017 | An FHIR-based Framework for Consolidation of Augmented EHR from Hospitals for Public Health Analysis | / | Analysis | Generic | Public Health/ Epidemiological Studies | 0 | 2017 IEEE 11th International Conference on Application of Information and Communication Technologies (AICT) | None | Yes | 0 | 0 | 0 | 0 | 0 | 1 |
| Khvastova et al. 2020 | Towards Interoperability in Clinical Research - Enabling FHIR on the Open-Source Research Platform XNAT | / | Data capture | Neuroimaging research | Clinical Research | 0 | Journal of medical systems | 3,18 | Yes | 0 | 0 | 0 | 0 | 0 | 1 |
| Kilintzis et al. 2022 | CoCross: An ICT Platform Enabling Monitoring Recording and Fusion of Clinical Information Chest Sounds and Imaging of COVID-19 ICU Patients. | / | Standardization of data | Infectious Disease | Clinical Research | 0 | healthcare | 1,58 | No | 0 | 0 | 0 | 0 | 1 | 0 |
| Klopfenstein et al. 2021 | Fast Healthcare Interoperability Resources (FHIR) in a FAIR Metadata Registry for COVID-19 Research. | FHIR® R4, v4.0.1 | Standardization of data | Generic | Research not specified | 0 | Studies in health technology and informatics | 0,678 | Yes | 0 | 0 | 0 | 0 | 0 | 0 |
| Lackerbauer et al. 2019 | Automated Verification of Structured Questionnaires Using HL7 | FHIR Standard 3.5.0, R4 | Recruitment | Generic | Research not specified | 0 | Studies in health technology and informatics | 0.68 | Yes | 0 | 0 | 0 | 0 | 0 | 1 |
| Lambarki et al. 2021 | Oncology on FHIR: A Data Model for Distributed Cancer Research. | R4 | Standardization of data | Oncology | Clinical Research | 0 | Studies in Health Technology and Informatics | 0,678 | Yes | 0 | 1 | 1 | 0 | 1 | 0 |
| Lee et al. 2020 | Global Infectious Disease Surveillance and Case Tracking Systemfor COVID-19:Development Study | R4 | Standardization of data | Infectious Disease | Clinical Research | 0 | JMIR Medical Informatics | 2,58 | Yes | 0 | 0 | 0 | 0 | 0 | 1 |
| Lenert et al. 2021 | Automated production of research data marts from a canonical fast healthcare interoperability resource data repository: applications to COVID-19 research. | FHIR version is 3.0.2; | Analysis | Infectious Disease | Research not specified | 0 | Journal of the American Medical Informatics Association | 4,497 | Yes | 0 | 0 | 0 | 0 | 0 | 1 |
| Leroux et al. 2017 | Towards achieving semantic interoperability of clinical study data with FHIR | / | Standardization of data | Generic | Clinical Research | 0 | Journal of biomedical semantics | 1,582 | Yes | 0 | 0 | 0 | 0 | 1 | 0 |
| Majeed et al. 2021 | HIStream-Import: A Generic ETL Framework for Processing Arbitrary Patient Data Collections or Hospital Information Systems into HL7 FHIR Bundles. | / | Standardization of data | Generic | Clinical Research | 0 | Studies in Health Technology and Informatics | 0,678 | Yes | 0 | 0 | 0 | 1 | 1 | 0 |
| Metke-Jimenez et al. 2019 | FHIRCap: Transforming REDCap forms into FHIR resources | / | Data capture | Genomics | Clinical Trials | 1 | AMIA Joint Summits on Translational Science proceedings | None | Yes | 1 | 1 | 0 | 0 | 1 | 0 |
| Peng et al. 2021 | Towards the Representation of Genomic Data in HL7 FHIR and OMOP CDM. | R4 | Standardization of data | Genomics | Clinical Research | 0 | Studies in Health Technology and Informatics | 0,678 | Yes | 0 | 0 | 0 | 1 | 0 | 0 |
| Pfiffner et al. 2016 | C3-PRO: Connecting ResearchKit to the Health System Using i2b2 and FHIR | DSTU 2 | Data capture | Infectious Disease | Clinical Trials | 1 | PLOS One | 2,74 | Yes | 0 | 0 | 0 | 0 | 0 | 1 |
| Reinecke et al. 2020 | Design for a Modular Clinical Trial Recruitment Support System Based on FHIR and OMOP | / | Recruitment | Generic | Clinical trials | 1 | Studies in health technology and informatics | 0.68 | Yes | 0 | 0 | 0 | 1 | 0 | 0 |
| Rinaldi et al. 2021 | Use of LOINC and SNOMED CT with FHIR for Microbiology Data. | R4 | Standardization of data | Infectious Disease | Research not specified | 0 | Studies in Health Technology and Informatics | 0,678 | No | 1 | 1 | 0 | 0 | 1 | 0 |
| Rinaldi et al. 2021 | From OpenEHR to FHIR and OMOP Data Model for Microbiology Findings | R4 | Standardization of data | Infectious Disease | Research not specified | 0 | Studies in Health Technology and Informatics | 0,678 | No | 1 | 1 | 0 | 1 | 1 | 0 |
| Sass et al. 2020 | The German Corona Consensus Dataset (GECCO): a standardized dataset for COVID-19 research in university medicine and beyond | R4 | Standardization of data | Infectious Disease | Clinical Research | 0 | BMC medical informatics and decision making | 2,317 | No | 1 | 1 | 1 | 1 | 1 | 0 |
| Sass et al. 2021 | Fast Healthcare Interoperability Resources (FHIR®) Representation of Medication Data Derived from German Procedure Classification Codes (OPS) Using Identification of Medicinal Products (IDMP) Compliant Terminology. | R4 | Standardization of data | Generic | Research not specified | 0 | Studies in Health Technology and Informatics | 0,678 | Yes | 1 | 0 | 0 | 0 | 1 | 0 |
| Tanaka et al. 2020 | Implementation of a Secured Cross-Institutional Data Collection Infrastructure by Applying HL7 FHIR on an Existing Distributed EMR Storages | / | Data capture | Generic | Clinical Research | 0 | Studies in health technology and informatics | 0.68 | Yes | 0 | 0 | 0 | 0 | 0 | 1 |
| Ulrich et al. 2016 | Metadata Repository for Improved Data Sharing and Reuse Based on HL7 FHIR | / | Standardization of data | Generic | Clinical Research | 0 | Studies in health technology and informatics | 0.68 | Yes | 0 | 0 | 0 | 0 | 1 | 0 |
| Wagholikar et al. 2017 | Evolving Research Data Sharing Networks to Clinical App Sharing Networks. | / | Data capture | Generic | Clinical Trials | 0 | AMIA Joint Summits on Translational Science proceedings | None | Yes | 0 | 0 | 0 | 0 | 1 | 0 |
| Wang et al. 2021 | Can FHIR Support Standardization in Post-Market Safety Surveillance? | / | Standardization of data | Generic | Clinical Research | 0 | Studies in Health Technology and Informatics | 0,678 | No | 0 | 0 | 0 | 0 | 0 | 1 |
| Weber et al. 2020 | A FHIR-Based eConsent App for the Digital Hospital | / | Consent management | Generic | Clinical trials | 1 | Studies in health technology and informatics | 0.68 | Yes | 0 | 0 | 0 | 0 | 0 | 1 |
| Wettenstein et al. 2021 | Feasibility Queries in Distributed Architectures - Concept and Implementation in HiGHmed. | R4 | Recruitment | Generic | Clinical Research | 0 | Studies in health technology and informatics | 0.678 | Yes | 0 | 0 | 0 | 0 | 1 | 0 |
| Wettenstein et al. 2021 | Data Sharing in Distributed Architectures - Concept and Implementation in HiGHmed. | R4 | Standardization of data | Generic | Research not specified | 0 | Studies in health technology and informatics | 0,678 | Yes | 0 | 0 | 0 | 0 | 1 | 0 |
| Wu et al. 2018 | SemEHR: A general-purpose semantic search system to surface semantic data from clinical notes for tailored care, trial recruitment, and clinical research | / | Data capture | Generic | Clinical Research | 0 | Journal of the American Medical Informatics Association | 4,46 | Yes | 1 | 1 | 1 | 0 | 1 | 0 |
| Xu et al. 2020 | FHIR PIT: an open software application for spatiotemporal integration of clinical data and environmental exposures data | / | Data capture | Environmental health | Public Health/ Epidemiological Studies | 0 | BMC medical informatics and decision making | 2,317 | Yes | 0 | 0 | 0 | 0 | 0 | 1 |
| Zong et al. 2020 | Developing an FHIR-Based Computational Pipeline for Automatic Population of Case Report Forms for Colorectal Cancer Clinical Trials Using Electronic Health Records. | / | Data capture | Oncology | Clinical Trials | 1 | JCO clinical cancer informatics | 1,224 | No | 1 | 1 | 1 | 0 | 0 | 0 |
| Zong et al. 2020 | Modeling cancer clinical trials using HL7 FHIR to support downstream applications: A case study with colorectal cancer data | FHIR Release 4 | Standardization of data | Oncology | Clinical Trials | 1 | International Journal of Medical Informatics | 3,59 | No | 0 | 0 | 0 | 0 | 0 | 1 |
| Zong et al. 2020 | Developing a FHIR-based Framework for Phenome Wide Association Studies: A Case Study with A Pan-Cancer Cohort. | R4 | Analysis | Genomics | Clinical Research | 0 | AMIA Joint Summits on Translational Science | None | No | 0 | 1 | 1 | 0 | 0 | 0 |
